# CpG Methylation Analysis of HPV16 in Laser Capture Microdissected Archival Tissue and Whole Tissue Sections from High Grade Anal Squamous Intraepithelial Lesions: A Potential Disease Biomarker

**DOI:** 10.1371/journal.pone.0160673

**Published:** 2016-08-16

**Authors:** Monica Molano, Sepehr N. Tabrizi, Suzanne M. Garland, Jennifer M. Roberts, Dorothy A. Machalek, Samuel Phillips, David Chandler, Richard J. Hillman, Andrew E. Grulich, Fengyi Jin, I. Mary Poynten, David J. Templeton, Alyssa M. Cornall, SPANC Study Team

**Affiliations:** 1 Regional HPV Labnet Reference Laboratory, Department of Microbiology and Infectious Diseases, The Royal Women’s Hospital, Parkville, Victoria, Australia; 2 Murdoch Children’s Research Institute, Parkville, Victoria, Australia; 3 Department of Obstetrics and Gynaecology, University of Melbourne and Murdoch Children’s Research Institute, Parkville, Victoria, Australia; 4 GynaePath, Douglass Hanly Moir Pathology, Macquarie Park, Sydney, Australia; 5 Australian Genome Research Facility, Perth, Australia; 6 Western Sydney Sexual Health Centre, Western Sydney Local Health District, Parramatta, New South Wales, Australia; 7 Sydney Medical School, Westmead, University of Sydney, Sydney, New South Wales, Australia; 8 The Kirby Institute, University of New South Wales, Sydney, New South Wales, Australia; 9 RPA Sexual Health, Royal Prince Alfred Hospital, Camperdown, Australia; Fondazione IRCCS Istituto Nazionale dei Tumori, ITALY

## Abstract

Incidence and mortality rates of anal cancer are increasing globally. More than 90% of anal squamous cell carcinomas (ASCC) are associated with human papillomavirus (HPV). Studies on HPV-related anogenital lesions have shown that patterns of methylation of viral and cellular DNA targets could potentially be developed as disease biomarkers. Lesion-specific DNA isolated from formalin-fixed paraffin-embedded (FFPE) tissues from existing or prospective patient cohorts may constitute a valuable resource for methylation analysis. However, low concentrations of DNA make these samples technically challenging to analyse using existing methods. We therefore set out to develop a sensitive and reproducible nested PCR-pyrosequencing based method to accurately quantify methylation at 10 CpG sites within the E2BS1, E2BS2,3,4 and Sp1 binding sites in the viral upstream regulatory region of HPV16 genome. Methylation analyses using primary and nested PCR-pyrosequencing on 52 FFPE tissue [26 paired whole tissue sections (WTS) and laser capture microdissected (LCM) tissues] from patients with anal squamous intraepithelial lesions was performed. Using nested PCR, methylation results were obtained for the E2BS1, E2BS2,3,4 and Sp1 binding sites in 86.4% of the WTS and 81.8% of the LCM samples. Methylation patterns were strongly correlated within median values of matched pairs of WTS and LCM sections, but overall methylation was higher in LCM samples at different CpG sites. High grade lesions showed low methylation levels in the E2BS1 and E2BS2 regions, with increased methylation detected in the E2BS,3,4/Sp1 regions, showing the highest methylation at CpG site 37. The method developed is highly sensitive in samples with low amounts of DNA and demonstrated to be suitable for archival samples. Our data shows a possible role of specific methylation in the HPV16 URR for detection of HSIL.

## Introduction

The incidence of anal cancer is increasing worldwide, in both females and males [1]. The vast majority are squamous cell carcinoma (SCC), which have a strong causal relationship with high risk human papillomavirus (HR HPV) [2, 3, 4]. High-grade squamous intraepithelial lesion (HSIL) is a precursor lesion and is very common in populations at high risk for anal cancer development [2, 5, 6]. Around 60% of the HSIL will become persistent and a small percentage of persistent HSIL will develop into cancer [7]. Although persistent infection with HR HPV has been associated with anal carcinogenesis, additional molecular events are required for malignant transformation. Abnormal DNA methylation is a well-recognized epigenetic hallmark of cancer cells and has been demonstrated to occur in many tumour types including HPV-associated cervical cancer [8]. The term DNA methylation refers to the introduction of methyl groups on cytosines located in CpG dinucleotides and leads to gene silencing after chromatin structure remodelling [9].

Studies of cervical cancer cell lines and cervical lesions have shown that regions of the HPV genome targeted for methylation have important roles in the viral life cycle and HPV associated oncogenesis [8, 9], and that patterns of methylation in these regions correlate with detection and persistence of high grade cervical lesions [9–11]. Although high levels of methylation of the HPV L1 gene have consistently been reported to be associated with neoplastic progression and detection of cervical HSIL [12–14], there are still conflicting results for the upstream regulatory region (URR) [15–18]. The role of HPV methylation in anal lesions is less well understood, with only one study examining patterns of methylation in the HPV enhancer and promoter regions in swabs of HIV positive men [19].

Methylation of CpG islands can be detected by different methods including methylation-specific PCR (MSP), quantitative methylation–specific PCR (qMSP), methylation-sensitive enzymes (COBRA assay), direct or next generation sequencing and pyrosequencing of bisulfite-modified DNA [11, 20–23]. The latter technique allows for relative quantitation of the base composition at each site sequenced. The ratio of C:T after bisulfite treatment indicates the proportion of unmethylated and methylated cytosines at each CpG site in the original sequence without the need for cloning [24–26].

DNA samples from formalin-fixed paraffin-embedded (FFPE) biopsy tissue obtained from epidemiological or clinical studies with known clinical follow-up are a valuable resource. These can be utilised as whole tissue sections (WTS), or after laser capture microdissection (LCM) has been performed to isolate lesion-specific tissue from surrounding biopsy material. Because these samples contain chemical contaminants and small quantities of DNA that is fragmented and degraded as a result of both formalin fixation and sodium bisulfite treatment, sensitive methods designed for detecting these amplicons are required. In this study, we developed and evaluated a sensitive, reproducible nested PCR-pyrosequencing based method for quantitative CpG site-specific methylation analysis of the URR of HPV16, comparing WTS with LCM tissue of patients with anal squamous intraepithelial lesions (ASIL).

## Materials and Methods

### Ethics

Ethics approval for the study was granted on 21 April 2010 by the St Vincent’s Hospital Ethics Committee in Sydney, NSW, Australia. File number 09/203. The study is conducted in accordance with the National Health and Medical Research Council (NHMRC) National Statement of Ethical Conduct in Human Research 2007 (http://www.nhmrc.gov.au/book/national-statement-ethical-conduct-human-research), the World Medical Association Declaration of Helsinki (October 2000) and the International Conference on Harmonisation Note for Guidance on Good Clinical Practice (ICH-GCP) (http://www.tga.gov.au/industry/clinical-trials-note-ich13595.htm). Written informed consent was obtained from all individuals before any study-specific procedures were performed.

### Patients and tissue samples

Anal squamous intraepithelial lesions (SIL) biopsies were taken during high-resolution anoscopy (HRA) from participants enrolled in the Study of the Prevention of Anal Cancer (SPANC). The SPANC study is an ongoing prospective cohort study based in Sydney, Australia. The study is exploring the epidemiology of anal HPV infection and related abnormalities among a community recruited cohort of HIV positive and negative gay and bisexual men [27]. Biopsy specimens were routinely formalin fixed and paraffin embedded (FFPE) [27, 28]. FFPE biopsies, previously determined by LCM and HPV genotype analysis to contain cervical lesions and be HPV16-positive were taken from 26 participants. DNA extraction on WTS and LCM was done as previously described [28]. Briefly, DNA from WTS was extracted using QIAampVR DNA FFPE Tissue kit (QIAGEN, Valencia, CA, USA) according to the manufacturer’s instructions, and eluted in 50μL of elution buffer. Lesion-specific tissue was isolated on the Veritas laser capture microdissection system (Arcturus Bioscience, Mountain View CA) as previously described [28]. DNA from LCM sections was extracted using the Arcturus PicoPure DNA extraction kit (Applied Biosystems, Foster City, CA, USA) as per protocol F of the PicoPure DNA Extraction Kit User guide. DNA was quantified using the Qubit dsDNA HS Assay kit (Life Technologies, Oregon, USA) on the Qubit 2.0 Fluorometer and stored at -20°C until use. In total, 52 samples (26 paired WTS and LCM samples, comprising 3 low-grade SIL (LSIL), 23 HSIL were analysed.

### Cell Lines

HPV16 positive cervical carcinoma cell lines CaSki (approximately 400 copies per cell of the HPV16 genome, ATCC CRL1550; American Type Culture Collection (ATCC), Manassas, VA, USA) and SiHa (1–2 copies of HPV16 per cell, ATCC HTB35; ATCC), previously characterised as having high and low CpG methylation levels respectively in the HPV16 URR [24], were used as methylation controls and during standardization of PCRs and pyrosequencing assays. DNA was extracted from cell lines on the MagNAPure 96 (Roche Diagnostics GmbH, Penzberg, Germany) using the DNA and Viral Nucleic Acid Small Volume Kit (Pathogen Universal 200 protocol).

### Bisulfite Modification

DNA extracted from cervical cell lines (100 ng) and from WTS and LCM samples (1ng-100ng) was bisulfite treated using Methylamp DNA modification Kit (Epigentek, Brooklyn, NY, USA) as per the manufacturer’s instructions. Modified DNA was eluted in 40μL of the Methylamp elution buffer.

### PCR amplification and pyrosequencing

**Fig 1:**
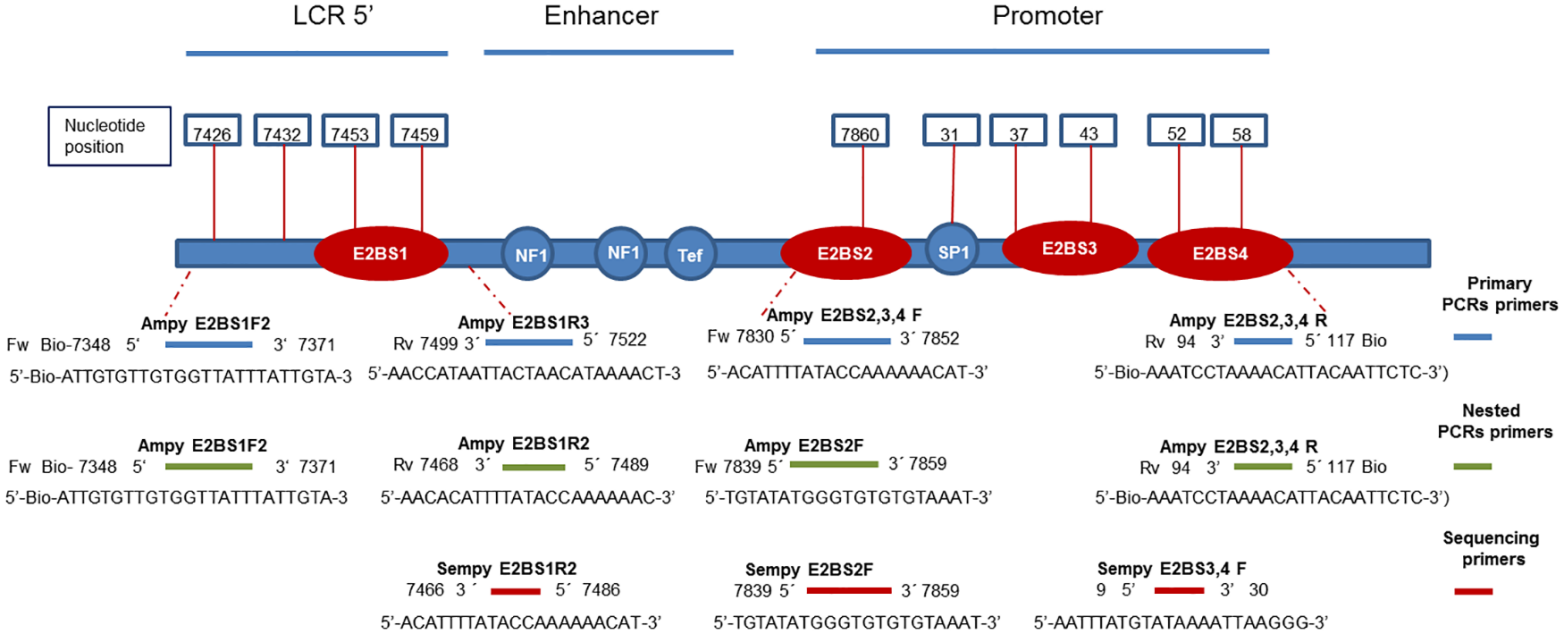
Schematic representation of the HPV16 URR including the 10 CpG sites studied and location of PCR primers.

Bisulfite modified DNA was amplified using four different sets of primers, in primary and nested PCR reactions, covering the E2BS1 binding site (4 CpG sites at positions 7426, 7432, 7453 and 7459) and the promoter region covering the E2BS2,3,4 and Sp1 binding sites (6 CpG sites at positions 7860, 31, 37, 43, 52 and 58) of the HPV 16 genome. Primers used for PCR amplification and pyrosequencing reactions have been modified from [24, 29] as previously described [28]. Fig 1 shows a schematic representation of the HPV16 URR, including the locations of the CpG dinucleotides and primers relevant to this study.

Primary PCR amplifications of the E2BS1 (175bp) and E2BS2,3,4 (193bp) regions were performed in two independent PCR reactions in a final reaction volume of 25μl each, containing 12.5μl of Hot Start Taq Master mix (Qiagen, Valencia, CA), 0.625μl of each 10μM forward and reverse primer (for the E2BS1 region, Ampy E2BS1 F2 and Ampy E2BS1 R3 primers; and for the E2BS2,3,4 region, Ampy E2BS2,3,4 F and Ampy E2BS2,3,4 R primers) (Fig 1), 5μl of bisulfite modified DNA and DNase/RNase free water. Thermocycler conditions were as follows: denaturation for 15 min at 95°C, followed by 50 cycles of amplification consisting of 40 sec at 95°C, 30 sec at 50°C, and 40 sec at 72°C, with a final extension of 6 min at 72°C.

A 1μl aliquot of each primary PCR product was included in each nested PCR amplification of the E2BS1 (142bp) and E2BS2,3,4 (184bp) regions. Each nested PCR reaction was performed using the same reaction mix as the primary PCR, with the exception of the E2BS1 reverse primer (Ampy E2BS1 R2) and the E2BS2,3,4 forward primer (Ampy E2BS2 F) (Fig 1). Thermocycler conditions were the same as those used for the primary PCR, except for the annealing temperature of the E2BS1 target which was increased to 56°C. Amplified products from primary and nested PCR reactions were analysed by 2.0% agarose gel electrophoresis, and only samples that produced a visible band were further analysed by pyrosequencing. A 20μl aliquot of each amplified product was used for pyrosequencing, which was carried out on the PyroMark Q24 instrument (Qiagen) at the Australian Genome Research Facility (AGRF) using the appropriate sequence primers [E2BS1 region (Sempy E2BS1 R2); E2BS2 region (Sempy E2BS2 F) and E2BS3,4 region (Sempy E2BS3,4 F)] in three separate sequencing reactions (Fig 1). Assay setup, sequence run, and analysis were performed with PyroMark Q24 Software. Bias tests of amplification were performed using methylated-unmethylated DNA dilution series (100%, 50%, 25%, 10% and 0% methylated target DNA in a background of unmethylated target DNA). Negative controls, with all of the reaction components but devoid of DNA, and methylation controls of bisulfite modified SiHa and CaSki DNA, were included in each experiment.

### Sensitivity and reproducibility of PCR amplification and pyrosequencing

To determine the limit of detection for primary and nested PCR amplification, tenfold serial dilutions of bisulfite modified SiHa DNA, from 10^5^ to 1 copy per reaction, were performed in triplicate, visualised on a 2% agarose gel and pyrosequenced. Theoretically 1ng of SiHa is equivalent to 10^3^ copies of HPV 16 DNA [30]. To determine the intra-assay reproducibility of quantitation of CpG methylation using nested PCR-pyrosequence analysis, aliquots of 100ng of SiHa and CaSki DNA were bisulfite modified and amplified in triplicate using the same conditions on the same day. Amplified products were then analysed by pyrosequencing. Inter-assay variability was measured by comparing aliquots of 100 ng of control DNA that were bisulfite modified, amplified and analysed by pyrosequencing on 4 separate days.

### Data analyses

Intra-assay repeatability and inter-assay reproducibility were assessed by calculating the mean, standard deviation (SD) and coefficient of variation (CV). Spearman correlation coefficient (r_s_) and corresponding p values were applied to assess the strength and significance of association between methylation levels in primary vs nested PCRs, and WTS vs LCM samples. The Wilcoxon matched pairs test was performed to compare methylation levels when necessary. Graphics and analyses were performed using Microsoft Office Excel 2010 and IBM SPSS Statistics 24 software (Chicago, IL, USA). All reported p values were two-sided and considered statistically significant if p ≤ 0.05.

## Results

### Improving the limit of detection of target amplification using a nested PCR

**Fig 2:**
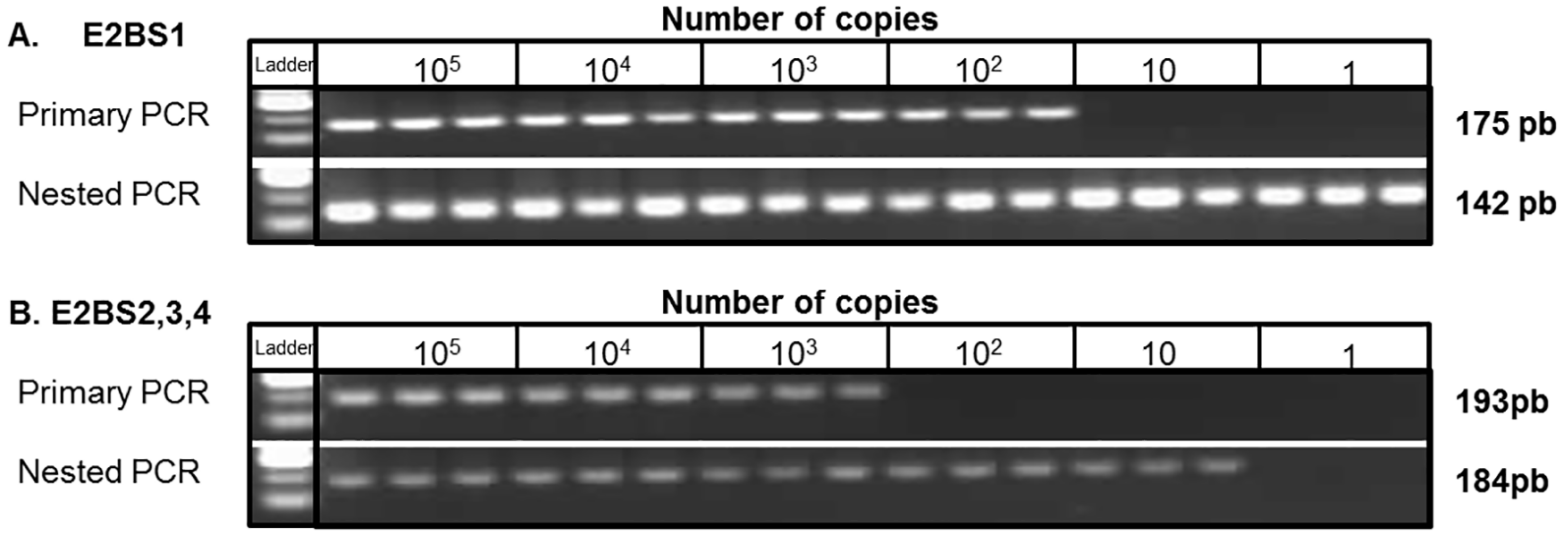
Detection limits for primary and nested PCRs. Serial dilutions of SiHa were subjected to primary PCR using specific primers for (A) E2BS1 region and (B) E2BS2,3,4 region of HPV16 in triplicate reactions. The primary PCR reactions were then used as templates to conduct nested PCR.

Amplification of bisulfite modified SiHa and CaSki DNA, using both primary and nested PCR, resulted in bands of the expected sizes when visualised on 2% agarose gel. Amplification of tenfold serial dilutions of bisulfite modified SiHa DNA produced visually detectable amplification products from a minimum of 100 copies for the E2BS1 primary primers and 1,000 copies for the E2BS2,3,4/Sp1 primary primers. Using the nested PCR primers, the limits of detection were improved by 10- to 100-fold, with visually detectable products amplified from a minimum of 1 copy and 10 copies for the E2BS1 and E2BS2,3,4/Sp1primer sets, respectively (Fig 2).

### Intra- and inter-assay reproducibility of pyrosequence analysis using controls

Intra-assay variability was low for all CpG sites, and ranged from 0–2.7% for CaSki, and 0–3.5% for SiHa. Inter-assay variability was also low for all CpG sites, ranging from 0.8–6.5% for CaSki and 0–3.9% for SiHa (Table 1).

**Table 1:** Inter-assay reproducibility and intra-assay repeatability of methylation at 10 different CpG sites of HPV16 URR in CaSki and SiHa cells.

			E2BS1				E2BS2	E2BS3,4				
			7459 CpG	7453 CpG	7432 CpG	7426 CpG	7860 CpG	31 CpG	37 CpG	43 CpG	52 CpG	58 CpG
**Inter-assay Reproducibility**		Mean	65.3	68.8	42.0	48.3	21.0	92.0	93.0	99.3	97.8	99.5
	**CaSki**	SD	2.8	3.1	2.7	3.0	1.2	1.6	1.2	1.3	2.3	0.9
		CV (%)	4.3	4.5	6.5	6.2	5.8	1.7	1.3	1.3	2.3	0.8
		Mean	3.5	1.8	6.8	2.8	1.0	1.0	2.3	1.3	2.0	2.3
	**SiHa**	SD	1.1	0.4	0.8	1.1	0.0	0.0	0.43	0.43	0.7	0.4
		CV (%)	3.1	2.4	1.2	3.9	0.0	0.0	1.9	3.4	3.5	1.9
**Intra-assay Repeatability**		Mean	65.3	67.3	40.3	46.36	21.36	93.0	93.7	100.0	97.3	100.0
	**CaSki**	SD	0.6	0.6	0.6	0.6	0.6	0.0	0.6	0.0	0.6	0.0
		CV (%)	0.9	0.9	1.46	1.26	2.7	0.0	0.6	0.0	0.6	0.0
		Mean	4.0	2.3	5.3	3.7	1.0	1.0	2.0	1.0	2.0	1.7
	**SiHa**	SD	0.0	0.6	0.6	0.6	0.0	0.0	0.0	0.0	0.0	0.6
		CV (%)	0.0	2.5	1.1	1.6	0.0	0.0	0.0	0.0	0.0	3.5

Four sequencing reactions were performed for the inter-assay reproducibility and for the intra-assay repeatability. Samples were tested in triplicate for each assay. SD = standard deviation. CV = coefficient of variation.

**Fig 3:**
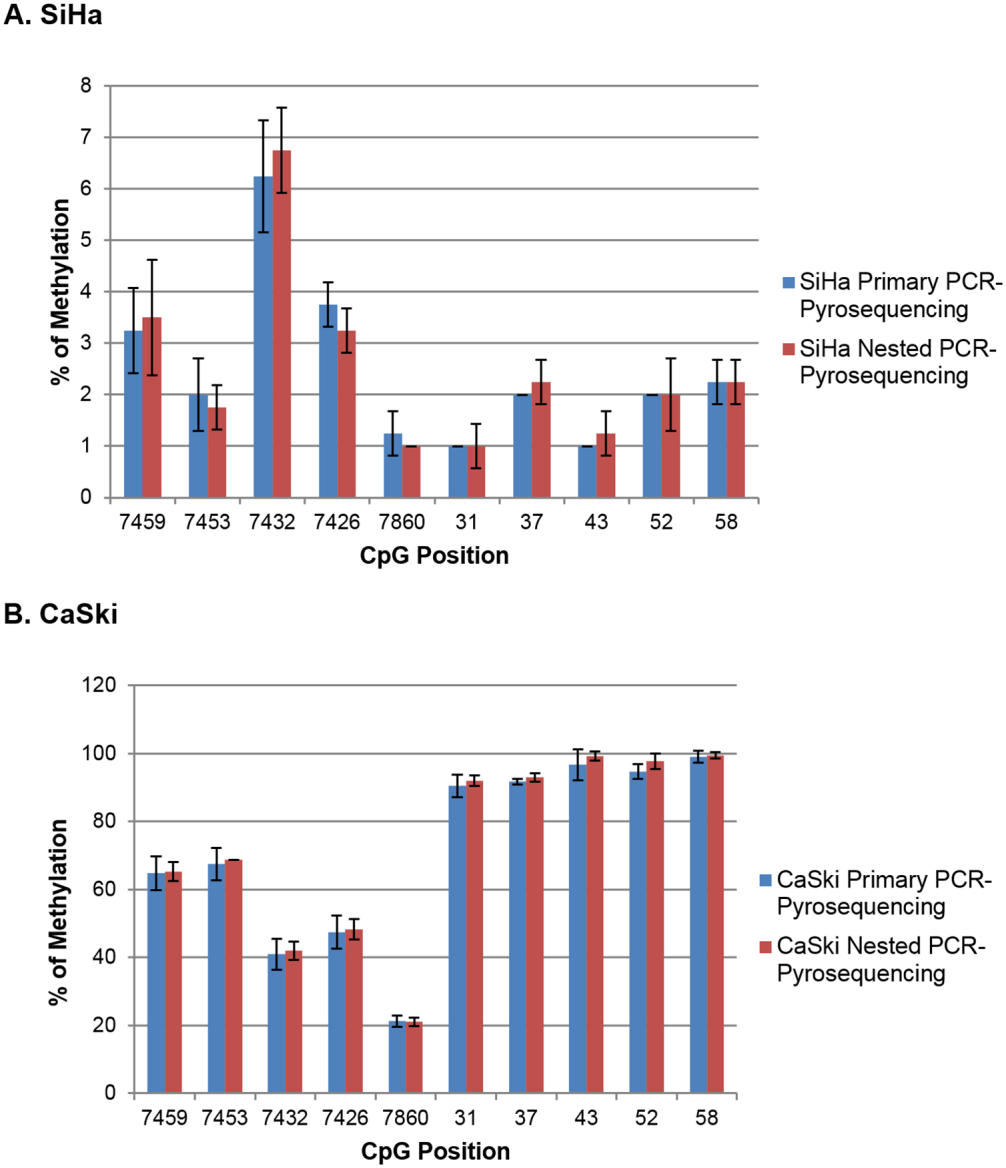
Comparison of methylation patterns by PCR type (Primary/nested) at 10 CpG sites of the URR of HPV 16 in (A) SiHa and (B) CaSki cells. The percentage of methylation for each CpG site are mean (±S.D) of four sequencing reactions. Coefficient of correlation between primary and nested PCRs were 0.986 and 0.999 in SiHa and CaSki cells respectively. Error bars indicate standard deviation.

**Fig 4:**
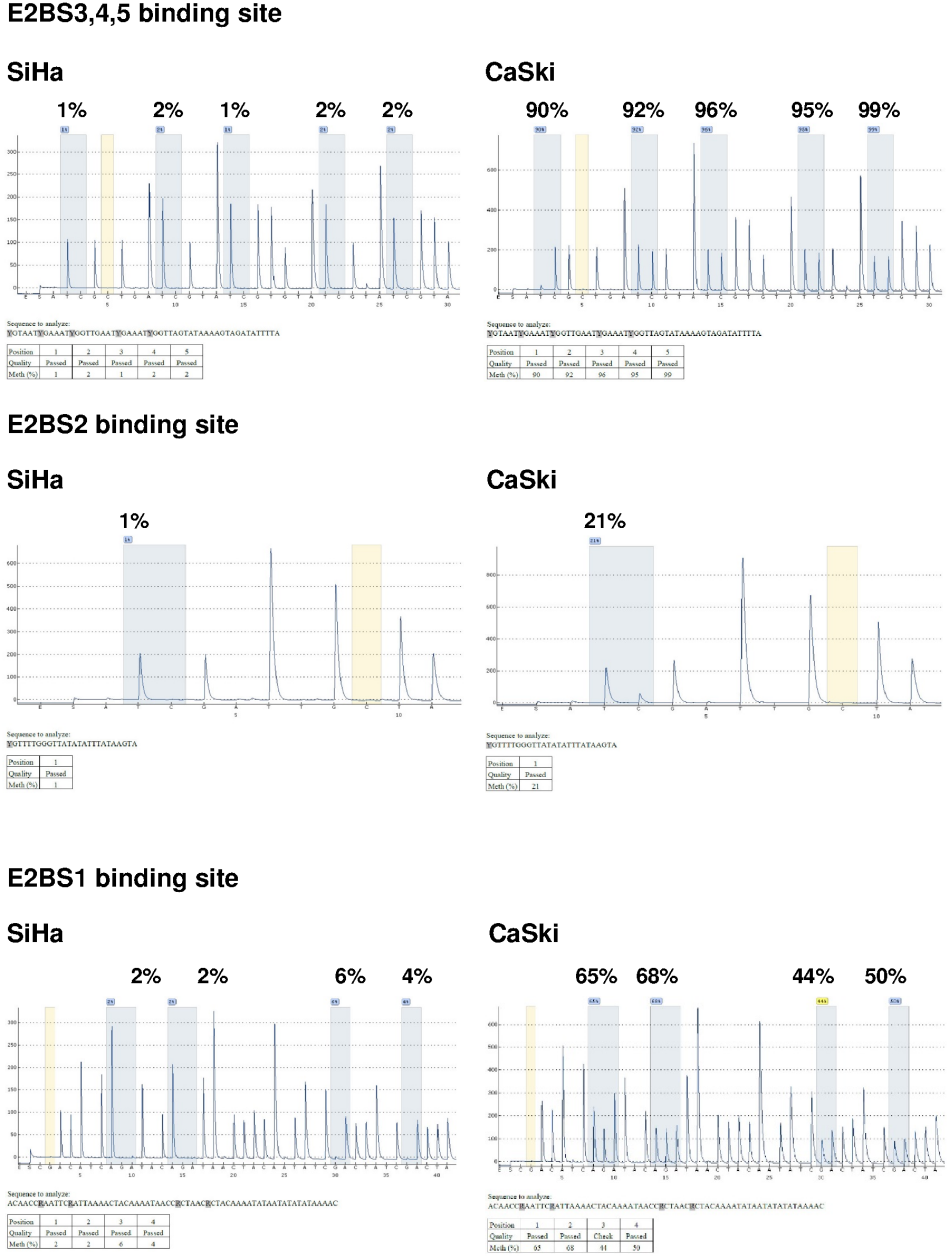
Representative pyrograms showing methylation levels at 10 CpG sites within the E2BS1, E2BS2,3,4 and Sp1 binding sites of HPV16 URR in SiHa and CaSki cells using nested amplified products.

Overall, SiHa was completely unmethylated (<10%) at all binding sites studied, while CaSki showed higher percentages of methylation with complete methylation (>90%) at binding sites 31, 37, 43, 52 and 58. Primary and nested PCRs showed similar percentages of methylation (Fig 3A and 3B) with a coefficient of correlation of r_s_ = 0.986, p<0.0001 and r_s_ = 0.999, p<0.0001 in SiHa and CaSki cells, respectively. Representative pyrograms showed methylation quantification at 10 CpG sites of HPV16 URR in SiHa and CaSki cells (Fig 4).

### Quantitation of CpG methylation from FFPE biopsy samples

From 52 samples analysed, 8 (15.4%) samples (4 WTS and 4 LCM) did not contain enough DNA for bisulfite modification (≥1ng) and methylation analysis. Table 2 shows the percentage of samples successfully amplified from the different regions using primary and nested PCRs in the remaining 44 samples (22 WTS and 22 LCM samples). Using the primary PCR, 40.9% of the WTS and 18.2% of the LCM samples amplified from the E2BS1, E2BS2,3,4 and Sp1 binding sites while using the nested PCR, 86.4% of the WTS and 81.8% of the LCM samples amplified from these binding sites.

**Table 2:** Percentage of amplification of the different regions using primary and nested PCRs in 44 samples.

AMPLIFIED REGION	TISSUE							
	WTS				LCM			
	Primary		Nested		Primary		Nested	
	n	%	n	%	n	%	n	%
**E2BS1 and E2BS2,3,4**	9	40.9	19	86.4	4	18.2	18	81.8
**Only E2BS1**	4	18.2	2	9.1	7	31.8	1	4.5
**Only E2BS2,3,4**	1	4.5	1	4.5	2	9.1	3	13.7
**No amplification**	8	36.4			9	40.9		
**Total**	22	100	22	100	22	100	22	100

**Fig 5:**
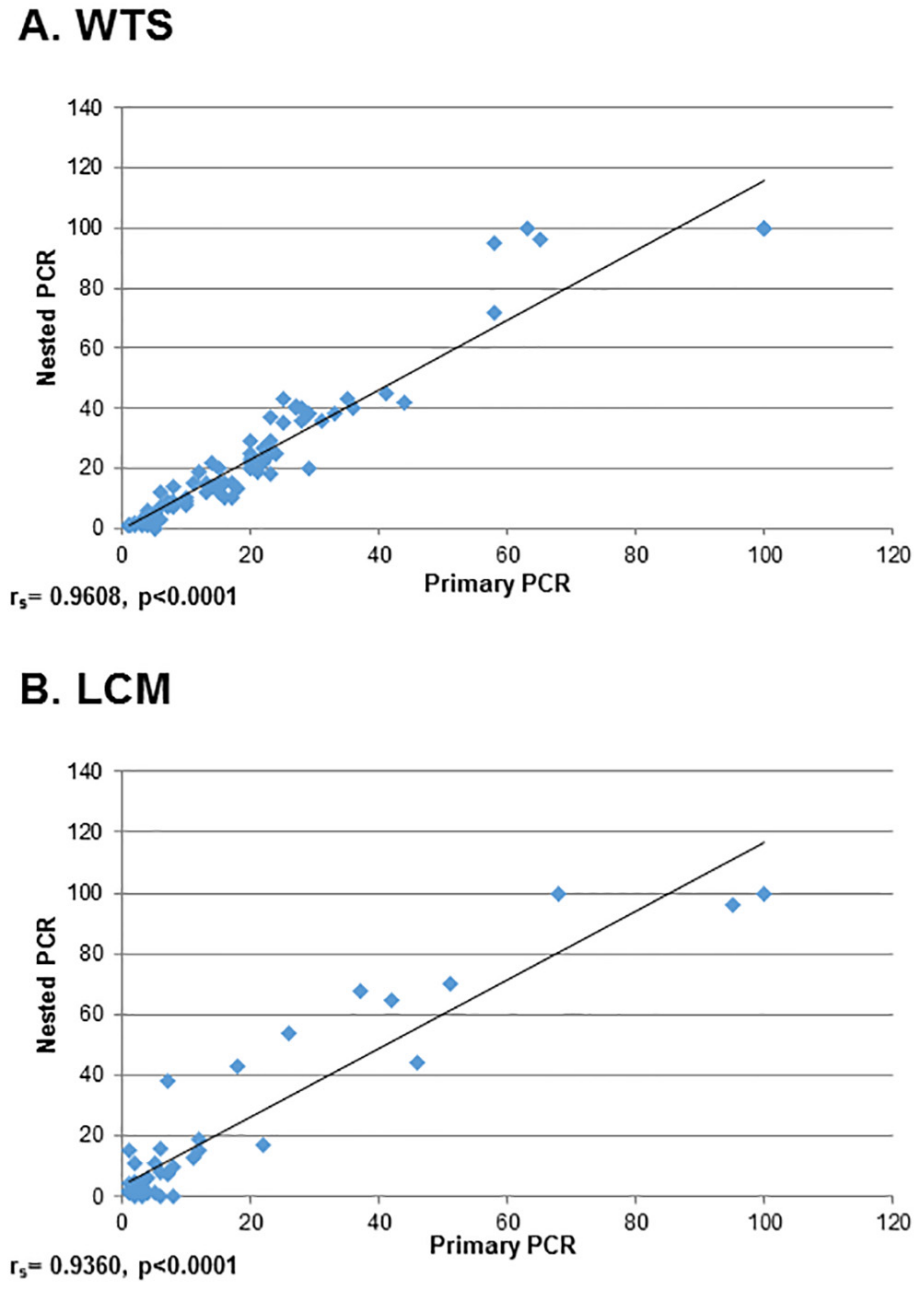
Correlation of methylation between primary and nested PCR in (A) WTS and (B) LCM samples. Correlations were determined using the Spearman rank correlation coefficient (r_s_). P ≤ 0.05 are considered significant.

Methylation results were strongly correlated between primary and nested PCRs. The coefficient of correlation between primary and nested PCR was r_s_ = 0.9608, p<0.0001 in WTS and r_s_ = 0.9360, p<0.0001 in LCM samples (Fig 5A and 5B).

**Fig 6:**
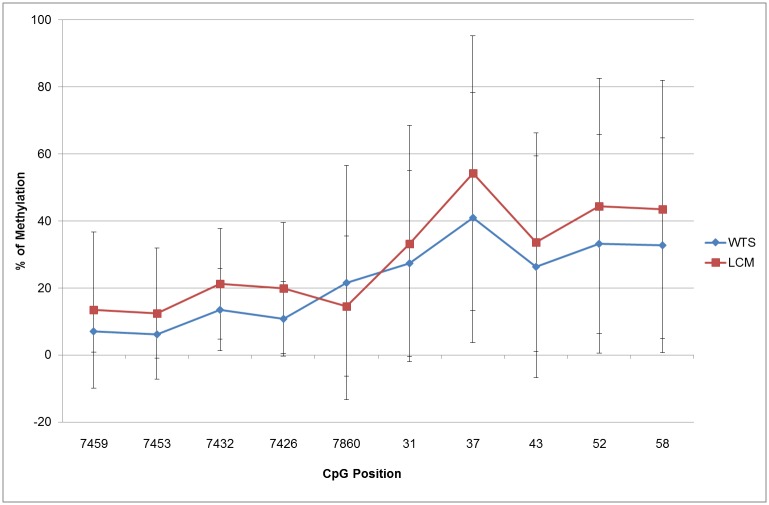
Pattern of methylation in 10 CpG sites of HPV16 URR using matched whole tissue sections (WTS) and laser capture microdissected (LCM) samples of patients with anal squamous intraepithelial lesions. Methylation levels at each CpG site represent mean of 14 subjects. Error bars indicate SD.

From the 22 WTS and 22 LCM samples that were analysed, 14 matched WTS/LCM pairs with high grade lesions amplified from all three regions (E2BS1, E2BS2,3,4 and Sp1 binding sites). Analyses of the pattern of methylation at 10 CpG sites comparing the mean values of these paired samples were strongly correlated, showing a coefficient of correlation of r_s_ = 0.937, p<0.0001. However, absolute values of methylation were higher in 9/10 CpG sites in the LCM samples compared with WTS (Fig 6). The profile of methylation along the regions at individual CpG sites showed low levels of methylation in the E2BS1 and E2BS2 binding sites and an increased level of methylation in the E2BS,3,4/ Sp1 binding sites, with the highest level of methylation at CpG site 37 followed by CpG site 52 and 58.

**Fig 7:**
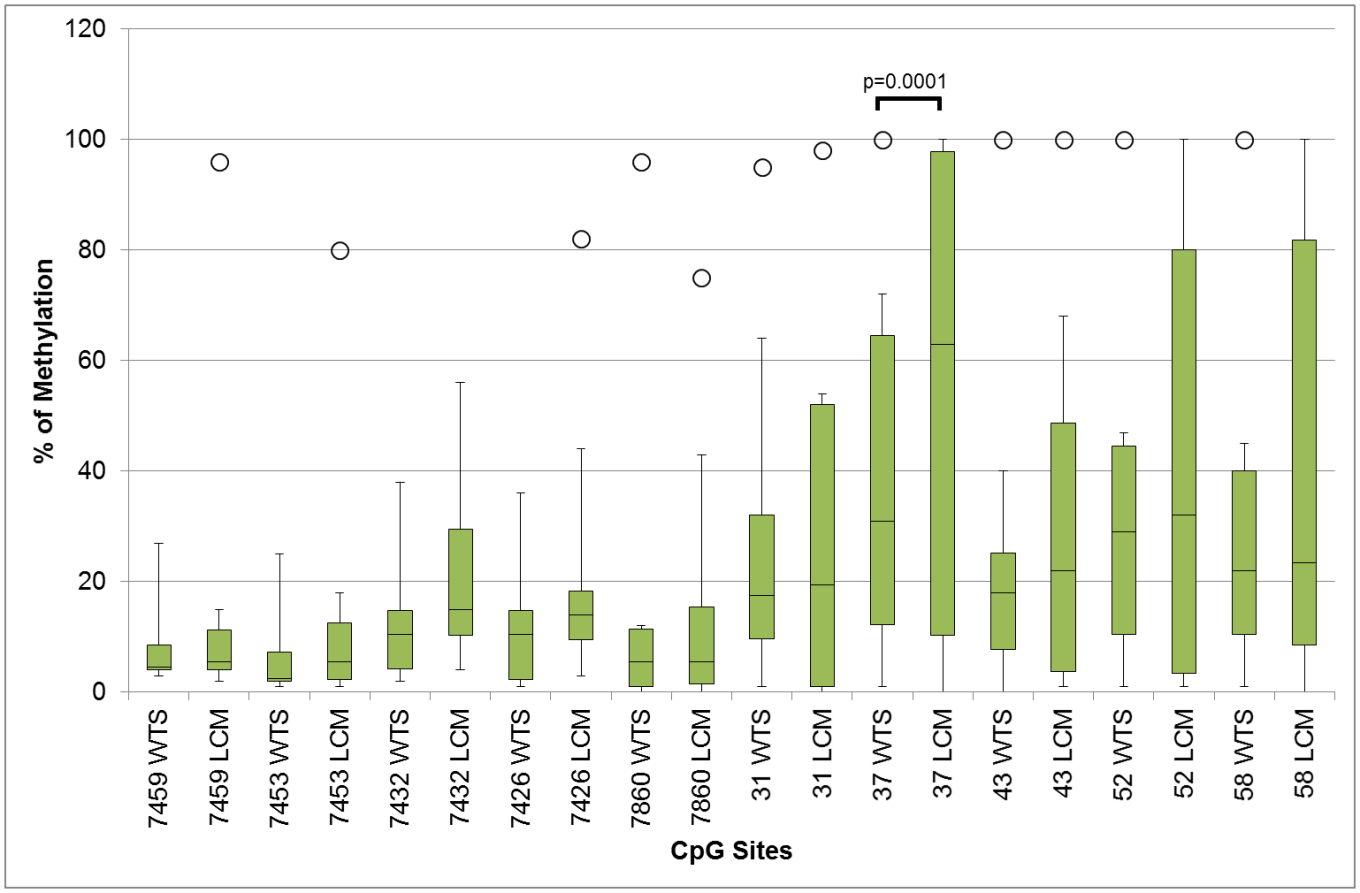
Analysis of methylation comparing median values between whole tissue section (WTS) and laser capture microdissected (LCM) tissue p value was derived from Wilcoxon matched pairs test comparisons. The central mark of the boxplots indicate the median, the edges mark of the box indicate the 25th and 75th percentiles and the whiskers indicate the minimum and maximum values. Individual points (circles) represent outliers.

Analysis of methylation comparing median values between paired WTS and LCM samples showed no significant differences in methylation levels for the majority of the CpG sites, but showed a higher percentage of methylation using LCM samples for some specific CpG sites of the E2BS2,3,4 region (Fig 7).

## Discussion

In this study we optimized a reproducible, site specific and quantitative method to analyse methylation status of 10 CpG sites within the URR of HPV16 in FFPE anal lesions. The method combines DNA bisulfite conversion, nested-PCR amplification and pyrosequencing of short amplicons. This method generated quantitative data from as little as 1–10 copies of HPV16 DNA from SiHa cell line and showed a high intra- and inter-assay reproducibility. To our knowledge, this is the first study that validates a nested PCR-pyrosequencing based method for the analysis of methylation of the URR of HPV16.

Quantitative results were similar to previous reports using primary PCR-pyrosequencing to quantify HPV 16 methylation in SiHa and CaSki cell lines [24, 29]. Using this method we were able to perform methylation analysis from WTS and LCM samples. We needed as little as 1 ng of DNA for optimal bisulfite modification. The nested PCRs used to amplify the E2BS1 and E2BS2,3,4/Sp1 binding sites were more sensitive than the primary PCRs alone, allowing methylation analysis in more than 80% of the samples. This higher level of detection may be in part due to the double amplification round, the smaller amplified product generated in the nested PCRs compared to the amplified products of the primary PCRs, and to a potential increase in efficiency of PCR amplification during the nested PCRs due to increased dilution of possible PCR inhibitors [31–32]. Reports from other researchers have indicated limited success analysing HPV methylation using LCM tissues, due to limited sensitivity of the assays used [20]. Recent reports of methylation analysis in archived FFPE samples, using nested PCR-pyrosequencing or nested methylation specific PCR have shown the advantages of these sensitive techniques for molecular epidemiology in a variety of other genes and diseases [33–35].

In this study the levels of methylation at individual CpG sites were very heterogeneous between HSIL samples. These results are similar to those reported by Wiley et al. that analysed the patterns of methylation in the enhancer and promoter segments of HPV16 URR in anal lesions in HIV positive men [19]. They are also similar to studies performed in cervical lesions [10, 11, 29]. In our study, the patterns of methylation using mean values of both LCM and WTS from patients with anal HSIL were similar throughout the E2BS1 and E2BS2,3,4/Sp1 binding sites of the URR, but the absolute level of methylation at each CpG site was higher when using LCM. The higher absolute level of methylation in LCM samples could reflect the more specific nature of the sample, comprising only lesion-specific cells, while the lower level of methylation in WTS is likely due to a mixture of lesion-specific cells and normal adjacent tissue, with heterogeneous epigenetic patterns.

In our study, the pattern of methylation in the E2BS1 and E2BS2 binding sites of patients with HSIL was low, increasing at the E2BS,3,4/Sp1 binding sites, showing the highest percentage of methylation at CpG site 37 followed by CpG site 52 and 58. These results are in agreement with a previous study of men with HSIL, which reported that methylation of at least one CpG site at nucleotides 31, 37, 43, 52, or 58 was associated with a 37-fold increase in the likelihood of HSIL (although the confidence interval was broad) [19]. These CpG sites could be used as promising markers in detection of HSIL. It has been shown that when E2 concentration is high, E2 binds to the E2 binding sites closest to the early promoter (E2BS2,3,4) blocking transcription from the early promoter [36–39]. Hence, when the E2 binding sites E2BS3 and E2BS4 are modified by methylation as was observed in this study, E2 is blocked from binding and therefore transcription of the E6 and E7 oncogenes is enhanced, thus facilitating development of neoplasia [26, 36, 39].

Conflicting results on the level of methylation in the URR have been reported in the literature, showing decreased methylation of CpG sites in some reports, and increased methylation in this region reported by others [10, 15, 16, 40–43]. These discrepancies may be explained by differences in the spectrum of lesions analyzed, techniques used in the methylation analysis, differences in the state of the viral genome within lesions, and differences in sampling methods. Some studies were conducted on fresh-frozen tissues or FFPE biopsy samples, whereas others were performed on exfoliated cervical cells [43]. Using LCM and WTS allows more precise analysis of the patterns of methylation in specific lesions as shown in our study, with a high correlation in the patterns of methylation between LCM and WTS in HSIL. In tissues with a higher mixture of lesions, the correlation of methylation between LCM and WTS has to be evaluated to decide if using methylation values of WTS will be enough to characterize the principal lesion found in the tissue or if LCM may still need to be performed to obtain more accurate information of the methylation status and for characterization of the lesion. In patients with advanced lesions and cancer that contain more homogeneous cell populations, the analysis of methylation will be less affected by differences in sampling, allowing the use of exfoliated cell specimens and other tissues for methylation. This could explain why methylation of the promoter region in cervical cancers is consistent in most of the studies, independent of the sample type analyzed [15, 37, 38, 44, 45]. The main purpose of this study was to validate an optimized method for quantitative analysis of methylation in anal biopsy samples. As a consequence, a limited number of samples were used and therefore further evaluation using a larger sample size is required to confirm association of methylation patterns with anal HSIL. However, the data obtained support a possible role and functional relevance of specific methylation in the HPV16 URR in anal HSIL, and therefore confirm the validity of the methodology used.

In conclusion, we have developed a sensitive and reproducible method for quantitative analysis of CpG methylation in the URR of HPV16 using DNA from FFPE anal biopsies. The use of LCM tissue was more targeted and provided a more accurate reflection of methylation in a lesion; however WTS was also a suitable sample type and could be utilized in larger studies. Our data shows the possible role and functional relevance of specific methylation in the HPV16 URR in anal HSIL. Analysis of methylation in this region will be used in the ongoing SPANC study to evaluate its utility in the detection of anal HSIL or as progression markers.
